# Factors associated with risk of malaria infection among pregnant women in Lagos, Nigeria

**DOI:** 10.1186/2049-9957-2-19

**Published:** 2013-08-30

**Authors:** Chimere O Agomo, Wellington A Oyibo

**Affiliations:** 1 ANDI Centre of Excellence for Malaria Diagnosis, International Malaria, Microscopy Training and RDT QA Programme, WHO/TDR/FIND Malaria Specimen Bank Site, Department of Medical Microbiology and Parasitology, College of Medicine, University of Lagos, Idi-Araba, Lagos, Nigeria; 2 Malaria Research Laboratory, Nigerian Institute of Medical Research, PMB 2013, Yaba, Lagos, Nigeria

**Keywords:** Malaria, Pregnancy, Risk factors, Insecticide spray, Maternal age, Malaria in pregnancy in Lagos

## Abstract

**Background:**

Pregnant women living in an area of stable malaria transmission such as Lagos, Nigeria, have been identified as being at an increased risk of the effects of malaria infection. In this area, most of the infections are asymptomatic which means they are overlooked and untreated much to the detriment of the mother and her foetus. The reality of scaled-up malaria interventions with long-lasting insecticide treated nets, vector control, artemisinin combination therapy (ACT) and intermittent preventive treatment of malaria pregnancy (IPTp) using sulphadoxine pyrimethamine (SP) is that it is also essential to determine the risk factors at play in these kinds of circumstances. This study was aimed at identifying the factors associated with risk of malaria infection in pregnant women in Lagos, Southwest Nigeria.

**Methods:**

Demographic information and malaria prevention practices of the pregnant women studied were captured using structured questionnaire. Microscopy was used to establish malaria infection, species identification and parasite density. Relative risk and multivariate logistic regression analysis were used to compare factors associated with malaria in pregnant women.

**Results:**

Malaria microscopy details, demographic information and malaria prevention practices of the pregnant women were obtained using a structured questionnaire. The prevalence of malaria using peripheral blood from 1,084 pregnant women that participated in the study was 7.7%. *Plasmodium falciparum* (*P. falciparum*) was seen in 95.2% of the cases as either mixed infection with *P. malariae* (3.6%) or as a mono infection (91.6%). Malaria preventive practices associated with a significant reduction (P<0.05) in the malaria infection was the use of insecticide sprays (RR = 0.36, 95 C.I. 0.24-0.54), and the combined use of insecticide spray and insecticide-treated nets (ITN) (RR= 6.53, 95% C.I. 0.92-46.33). Sleeping under ITN alone (RR = 1.07, 95% C.I. 0.55-2.09) was not associated with significant reduction in malaria infection among the study participants with malaria parasitaemia. Young maternal age (<20years) (RR = 2.86, 95% C.I. 1.48 – 5.50), but not primigravidity (RR = 1.36, 95% C.I. 0.90-2.05), was associated with an increased risk of malaria infection during pregnancy. After a multivariate logistic regression, young maternal age (OR = 2.61, 95% C.I. 1.13 – 6.03) and the use of insecticide spray (OR = 0.38, 95% C.I. 0.24-0.63) were associated with an increase and a reduction in malaria infection, respectively.

**Conclusion:**

Malaria prevalence was low among the pregnant women studied. Young maternal age and non-usage of insecticidal spray were the main factors associated with an increased risk of malaria infection among pregnant women in Lagos, Nigeria.

## Multilingual abstracts

Please see Additional file 1 for translations of the abstract into the six official working languages of the United Nations.

## Background

Malaria control still remains a challenge in Africa where 45 countries, including Nigeria, are endemic for malaria, and about 588 million people are at risk [1]. The protection of pregnant women living in malaria endemic countries has been of particular interest to many National Malaria Control Programmes because of the reduction in immunity associated with pregnancy. The reduction in cell-mediated immunity is a physiological response that allows foetal allograft retention, but it is thought to also interfere with resistance to various infectious diseases [2].

In malaria endemic regions, individuals are constantly exposed to malaria parasites through the bites of infected female *Anopheles* mosquitoes. This frequent exposure leads to the development of an effective anti-disease immunity to malaria, which prevents life-threatening parasite burdens and suppresses the pro-inflammatory responses which cause illness [3]. During pregnancy, the acquired semi-immunity is able to keep the infection at an asymptomatic level in the majority of cases [4-6]. Depending on the endemicity of malaria in an area, it can be expected that 1-50% of pregnant women may carry malaria parasitaemia, especially in the placenta, without noticing it [7,8]. At this period, unfortunately, the subclinical infection still poses a great danger to both the mother and the foetus. The principal impact of malaria infection in pregnancy is due to the presence of parasites in the placenta causing maternal anaemia (potentially responsible for maternal death when severe) and low birth weight (LBW), a major predictor of infant and neonatal mortality [9,10].

The focus of malaria prevention during pregnancy has been the use of antimalarial chemoprophylaxis and the use of insecticide treated nets (ITNs). Pregnant women on antimalarial chemoprophylaxis are at a reduced risk of the harmful effects of malaria [11], while ITNs reduce human contact with mosquitoes leading to a significant reduction in the incidence of malaria, severe morbidity and mortality due to malaria, as well as helping reduce the adverse effects of malaria during pregnancy in an area of intense malaria transmission [12-15].

In the World Malaria Report 2012 [1], Nigeria accounted for a quarter of all malaria cases in the 45 countries endemic for malaria in Africa. In Nigeria, 11% of maternal deaths are attributed to malaria [16]. However, malaria prevention measures have received great attention in the last six years as increased funding has resulted in the scale-up of malaria control efforts. The reports on the prevalence of malaria in pregnancy in different regions of Nigeria ranged from 19.7% to 72% [17-20]. Nevertheless, the method employed in any diagnosis is an important criterion in reporting valid results. The accuracy of a malaria microscopy result is influenced by factors such as training and retraining, experience, motivation and laboratory facilities [21,22].

The paucity of data on the risk factors associated with malaria in pregnancy with the current reality of massive malaria interventions necessitated this study, as a first step, in providing focused intervention in malaria during pregnancy, whilst also employing high-quality malaria microscopy in reporting malaria in pregnancy given the varied reports in Lagos, Southwest Nigeria.

## Methods

### Study area and participants

A total of 1,084 pregnant women who were attending antenatal clinics (ANC) for the first time during current pregnancy in two hospitals in semi-urban areas of Lagos – Ajeromi General Hospital in Ajegunle (501) and St Kizito Primary Health Center in Lekki (583) – and who consented to the study were enrolled between March 2007 and February 2008. These antenatal clinics were chosen because they record high antenatal clinic attendance. In sub-Saharan Africa, high antenatal clinic visits are common [23]. The antenatal clinics met this criterion of providing four qualitative visits by pregnant women. Since adequate malaria control strategies were expected to be initiated after booking at antenatal clinics, the women were recruited on the first day of registration for ANC.

This study was approved by the Ethics and Experimental Committee of the College of Medicine of the University of Lagos Lagos, Nigeria. All participants gave informed consent after the purpose of the research was explained to them. Participation in this research was voluntary as participants could decline to participate or withdraw from participation even after giving consent at any stage of the research. Patients that declined to participate were not denied access to the available routine care.

### Data collection and laboratory analysis

The study design was cross-sectional. The minimum study population was 384 as estimated with the Statcalc software of Epi Info 6 (Center for Disease Control, Atlanta) for Population Survey or Descriptive Study using random sampling. The sample size was calculated using the following assumptions: the population size of Lagos based on the national census was 9,013,534; the population of pregnant women in 2006 was 396,595 (4.4% of the Lagos population) (UNFPA http://www.unfpa.org/emergencies/manual/9a5.htm. Accessed 19th September 2006); the population of pregnant women in 2007 (growth of 2.34%) was 405,875; expected frequency of malaria in pregnancy of 10%; worst acceptable frequency of malaria in pregnancy of 7.0% and a confidence level of 95%. However, the total number of pregnant women recruited in the study was 1,084.

Demographic data, and information on history of fever during current pregnancy, treatment and preventive measures adopted were collected from the pregnant women using a semi-structured questionnaire which was administered by a trained interviewer. Peripheral blood by venepuncture was used to collect 1mL of blood for malaria microscopy and packed cell volume determination.

The total leukocyte count determination was done using the improved Neubauer Chamber, as described in detail by Baker and Silverton [24]. Briefly, 20 μL taken with an adjustable micropipette (P20 Pipetman, Gilson) of blood was mixed with 380 μL of Turk’s solution (2% Acetic acid tinged with gentian violet) taken with an adjustable micropipette (P1000 Pipetman, Gilson) to give a final 1:20 dilution. The red cells were lysed leaving the leukocytes. The leukocytes were counted using the New Improved Neubauer Chamber.

The haematocrit level was determined by filling a capillary tube (Hawksley, England) up to 75% with well-mixed anticoagulated blood, sealed at one end with Cristaseal, (Hawksley, England) and spun at 13,000 rpm for five minutes in a Hawksley haematocrit centrifuge (Hawksley, England). The percentage of the packed cells was read with a Hawksley Haematocrit Reader (Hawksley, England).

### Malaria microscopy

Thin and thick blood films for the diagnosis of malaria were prepared on the same slide. For the thick film, 12 μL of blood was taken with an adjustable micropipette (P20 Pipetman, Gilson) and spread over a diameter of 15 mm, while 2 μL of blood was used to make the thin film. The slides were made in duplicates and labeled appropriately. The thin film end of the slide was fixed by dipping the prepared film in absolute methanol for one to two seconds, and both films were allowed to dry for 24–48 hours and subsequently stained with 3% Giemsa at pH 7.2 for 45 minutes.

The stained slides were read by two certified microscopists. Discrepant parasite detection and parasite count readings between the two microscopists were resolved by a re-reading of the slides or by employing a third microscopist before the final result was determined. The mean parasite counts of the two readers were accepted if the discrepancy of the two readings was less than 20%. A slide was confirmed negative when 100 high power fields have been examined under x100 oil immersion objective lens.

The absolute parasite density was calculated using the formula: [# of parasites counted x total leukocyte count] / # of leucocytes counted.

### Data analysis

The data generated from the study were analysed using EPI INFO 2002 statistical software (CDC, Atlanta, USA). Tests for associations and differences were done by chi-square analysis, risk ratio analysis, Fisher Exact test, analysis of variance and a Kruskal-Wallis test where appropriate. Test of statistical significance was set at p value less than 0.05 at 95% confidence interval.

## Results

A total of 1,084 pregnant women participated in the study at their first antenatal clinic care (ANC) visit at either the St Kizito Primary Health Center (583) or Ajeromi General Hospital (501). The demographic characteristics of the pregnant women that participated in this study are summarised in Table 1. The mean age was 27.4±4.8 years (range, 15–42), and over half – 567 (52.3%) – were in their second trimester. Almost two-thirds of the pregnant women (64.1%) had secondary education. The occupation of the study participants included: trading, 359 (33.1%); full-time housewives, 317 (29.2%) and artisans, 189 (17.4%). There were 161 women that used a bed net (14.9%) (any type), while 125 (11.5%) used insecticide-treated nets.

**Table 1:** Demographic characteristics of pregnant women that attended the antenatal clinics in Lagos, Nigeria

**Description**	**No. (%)**
**Age group (years)**	
15-19	39 (3.6)
20-24	266 (24.5)
25-29	434 (40.0)
30-34	261 (24.1)
>34	84 (7.7)
**Occupation**	
Artisan	189 (17.4)
Trader	359 (33.1)
Civil servant	50 (4.6)
Housewife	317 (29.2)
Professional	24 (2.2)
Student	91 (8.4)
Teacher	54 (5.0)
**Education**	
Primary	103 (9.5)
Secondary	694 (64.1)
Tertiary	286 (26.4)
**Gravidity**	
Primigravidae	419 (38.7)
Secundigravidae	271 (25.0)
Multigravidae	393 (36.3)
**Trimester**	
1^st^	235 (21.7)
2^nd^	567 (52.3)
3^rd^	274 (26.0)
**Bed nets**	
Any net (possession)	325 (30.0)
Any net (use)	161 (14.9)
ITN (possession)	230 (21.2)
ITN (use)	125 (11.5)

**Figure 1:**
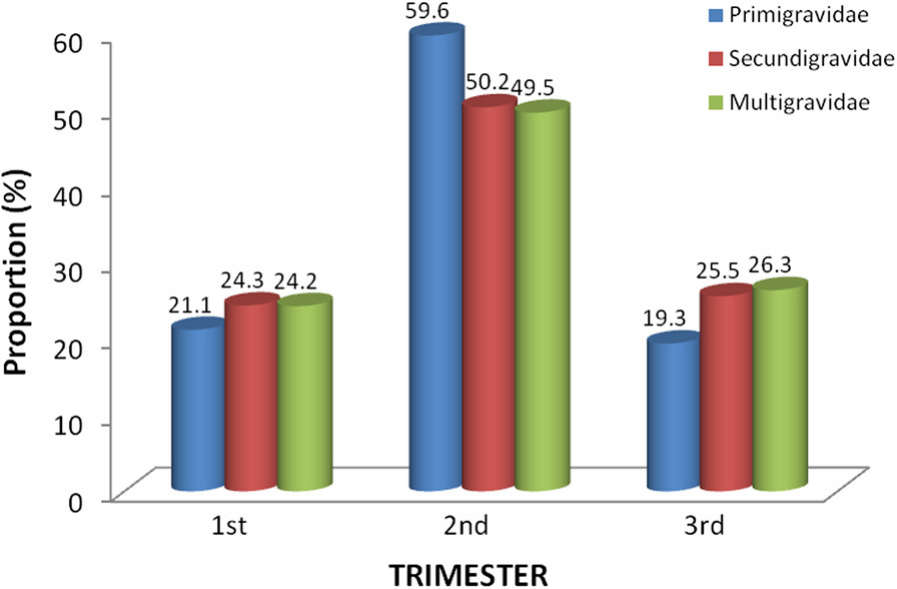
The pattern of booking at antenatal clinics by pregnant women based on gravidity and gestation age.

The pattern of first registration at the antenatal clinics showed that majority of the participants of all gravidities presented at the second trimester (P = 0.698) (see Figure 1). Thirty-three (84.6%) of the pregnant women in the age group, 15–19 years were primigravidae, while 71 (84.5%) of those in age group >34 years were multigravidae (see Table 2). There was a direct correlation (r = 0.54) between age and gravidity.

**Table 2:** Comparison of mean parasitaemia with age of the pregnant women studied

**Age group (years)**	**Malaria infection (%)**	**Geometric Mean Parasitaemia (range)**
15-19	8 (20.5%)	1457 (38–15,926)
20-24	26 (9.8%)	685 (40–54,000)
25-29	29 (6.7%)	383 (16–125,224)
30-34	14 (5.4%)	144 (11–54,000)
>34	6 (7.1%)	93 (41–141)
**Total**	83 (7.7)	395 (11–125,224)
P	0.010	0.041

The *Plasmodium* species detected in the positive malaria slide of pregnant women whose peripheral blood smear was tested were: *P. falciparum* 76 (91.6%), *P. malariae* 4 (4.8%), and mixed infection of *P. falciparum* and *P. malariae* 3 (3.6%).

Malaria prevalence was 7.7% (n=83). The age of the women was significantly associated with malaria prevalence (P=0.010) and parasite density (P=0.04). The 15–19 years age group had the highest prevalence (20.5%) as well as the highest geometric mean parasite density (1,457 parasites/μL of blood) (see Table 2).

The gravidity of the women was not associated with either malaria prevalence or mean geometric parasite density (P>0.05). The prevalence of malaria in primigravidae, secundigravidae and multigravidae were: 9.1%, 7.1% and 6.5%, respectively (P= 0.333). The geometric mean parasitaemia in the primigravidae, secundigravidae and multigravidae were 561 (range: 16–54,000), 255 (range: 25–125,224) and 305 (range: 11–54,000) parasites/μl of blood respectively (P = 0.346).

There was no significant association between malaria prevalence and either education (P= 0.215) or the gestational age at the time of booking (P = 0.577). Malaria prevalence in women with primary, secondary and tertiary education was 11.7%, 7.6% and 6.3%, respectively; while the prevalence in women that booked in ANC at the first, second and third trimesters were 6.8%, 8.5% and 6.7%, respectively.

Young maternal age (<20 years) was significantly associated with an increased risk of malaria infection (RR =2.86, 95% C.I. 1.48-5.50), however primigravidity and low educational status were not associated with increased risk of malaria infection (see Table 3).

**Table 3:** Relative risk of malaria infection in relation to demographic characteristics of pregnant women attending antenatal clinics in Lagos, Nigeria

**Character**	**Proportion with malaria**	**Risk ratio (95% C.I.)**	**P**
**Young age (<20 years)**			
Yes	8/39 (20.5%)	2.86 (1.48-5.50)	0.007
No	75/1045 (7.2)		
**Primigravidity**			
Yes	38/416 (9.1%)	1.36 (0.90-2.05)	0.093
No	45/668 (6.7%)		
**Low education level**^**ф**^			
Yes	65/798 (8.1%)	1.29 (0.78-2.14)	0.190
No	18/286 (6.3%)		

^ф^ Low education level = Secondary education or below.

Malaria preventive methods which were associated with a reduced risk of malaria infection were the use of insecticide spray (RR=0.36, 95% C.I. 0.24-0.54; P < 0.001), and the combined use of insecticide spray and ITN (RR=0.15, 95% C.I. 0.02-1.09; P = 0.011). The use of >ITNs alone was not significantly associated with a reduction in malaria infection (RR= 0.93 95% C.I. 0.48-1.82; P = 0.506). None of the women that used a combination of insecticide spray, ITN and chemoprophylaxis had malaria parasitaemia (see Table 4).

**Table 4:** Relative risk of malaria parasitaemia in relation to malaria prevention methods adopted by pregnant women attending antenatal clinics in Lagos, Nigeria

**Preventive methods**	**Proportion with malaria**	**Risk ratio (95% C.I.)**	**P**
**a) Insecticide spray**			
Yes	51/885 (5.8%)	0.36 (0.24–0.54)	<0.001
No	32/199 (16.1%)		
**b) Chemoprophylaxis**			
Yes	21/243 (8.6%)	1.17 (0.73–1.88)	0.297
No	62/841 (7.4%)		
**c) Bed net use**			
i) Any net			
Yes	9/161 (5.6%)	0.70 (0.36–1.36)	0.183
No	74/923 (8.0%)		
ii) ***Insecticide treated net (ITN)***			
Yes	9/125 (7.2%)	0.93 (0.48–1.82)	0.506
No	74/959 (7.7%)		
**d) Insecticide sprays + ITN**			
Yes	1/80 (1.3%)	0.15 (0.02–1.09)	0.011
No	82/1004 (8.6)		
**e) Chemoprophylaxis + ITN**			
Yes	1/22 (4.5%)	0.59 (0.09–4.04	0.488
No	82/1062 (7.7%)		
**f) Insecticide sprays + chemoprophylaxis**			
Yes	14/190 (7.4%)	0.95 (0.55–1.66)	0.505
No	69/894 (7.7%)		
**g) Insecticide sprays + Chemoprophylaxis + ITN**			
Yes	0/11 (0%)	-	-
No	83/1073 (7.7%)		

After adjusting for possible confounders, the use of insecticide spray (P<0.001) and young maternal age (P = 0.020) were the main factors associated with a reduced and an increased risk of malaria infection among pregnant women in Lagos, respectively (see Table 5).

**Table 5:** Multivariate comparison of factors associated with malaria infection in pregnant women in Lagos, Nigeria

**Variable**	**Odds Ratio**	**95% C.I.**	**P**
ITN + insecticide spray (Yes/No)	0.19	0.03-1.37	0.099
Insecticide spray (Yes/No)	0.37	0.23-0.59	<0.001
Young maternal age <20 years (Yes/No)	2.7	1.17-6.30	0.020

## Discussion

The *Plasmodium* species seen in this study confirm a previous report that *P. falciparum* is the most prevalent species in Nigeria accounting for about 98% of malaria cases in the country [25]. Steffen *et al.*[26] reported that 80-95% of malaria infections in tropical Africa are caused by *P. falciparum*.

Until now, the reports of prevalence of malaria in pregnancy have been very high, especially in Southwest Nigeria where prevalence rates of between 34.0% and 72% [4,17,19,20] have been reported. These reports contrast sharply with our finding in this same region among pregnant women attending antenatal clinics for the first time during their current pregnancy. The large differences in the reported prevalence rates of malaria may be attributed to skill and experience of laboratory personnel involved in blood film preparation, and the staining and reading of the slides. This was evidenced by analysed results of pre-tests conducted in 2010 on malaria microscopists that showed very low sensitivity of their capacity to accurately report malaria blood smears correctly (Oyibo *et al.,* unpublished data). In our study, blood smears were examined by microscopists whose sensitivity and specificity were above 90%. Strict adherence to procedures for slide preparation and staining [27] ensured the production of clear, well-stained slides, thereby reducing errors due to artefacts. The inaccurate diagnosis of malaria is not peculiar to Nigeria. In Tanzania, Mwanziva *et al.*[5] reported a case where about 99% of malaria positive slides from a Tanzanian clinic were actually negative.

The consequences of over-reporting of malaria cases are: (1) difficulty in assessing the impact of malaria control programmes due to baseline information before implementation sometimes not being accurate; (2) unnecessary treatment with antimalarial drugs for febrile cases not due to malaria, thereby, increasing the chances of developing antimalarial drug resistance; and (3) inaccurate national and, therefore, world malaria statistics. Situations such as these may require a total re-evaluation of reports indicating that over half of the cases of malaria in Africa are in Nigeria, the Democratic Republic of the Congo, Ethiopia, United Republic of Tanzania and Kenya [1].

While acknowledging the likely over-reporting of malaria in published reports, it is important to also note that malaria prevalence has been shown to be on the decline in several malaria endemic countries following the scaling-up of malaria interventions including: increased coverage in the distribution of Long Lasting Insecticide Treated Nets (LLINs), use of effective ACTs, indoor residual spraying and the institution of environmental management in some settings [1]. In Lagos, malaria control has been scaled up in the last ten years with the ‘EKO Free Malaria Programme’ that targeted pregnant women and children less than five years of age. Funding for malaria intervention and attention has also increased in the last six to eight years. Furthermore, the transmission of malaria in the catchment areas of the health facilities studied may be low given their urban nature though reports of malaria in pregnancy from rural/peri-urban area of Lagos are not available. All of these may have accounted for the prevalence reported in this study.

### Relation to age and gravidity

In this study, maternal age was associated with malaria prevalence, showing that a pregnant woman of young maternal age is at the greatest risk of malaria infection, as well as having the highest parasite densities. Similar findings have been reported by other authors in Gabon and Eastern Sudan where malaria prevalence was observed to decrease as age increased [28,29].

The effects of malaria in pregnancy have been noted to be lower in multigravidas than in other gravidities as a result of acquisition of specific immunity to placental malaria due to previous exposure [6,14,30]. Acquired specific immunity accumulates with subsequent infection and subsequent pregnancies [14]. In this study, gravidity was not associated with either malaria prevalence or the level of parasitaemia. Young maternal age has been reported as a more important risk factor than gravidity [31] and is consistent with this study. The level of acquired immunity, which is associated with the number of malaria infections during pregnancy, was not determined in this study. If this was done, it could have explained the lack of association between malaria prevalence and gravidity. In a malaria endemic area such as Lagos, it is possible that the women would have had a number of encounters with malaria infection prior to booking at the antenatal clinics used in this study. It could be that there was no difference in the level of specific immunity of the study participants based on gravidity. Moreover, some authors have also observed that women in their second pregnancies are almost as susceptible to malarial infections as those that are pregnant for the first time [7,32]. Early attendance and participation in focused ANC services is recommended to all pregnant women especially the primigravidae so as to reduce the risk of malaria infection in pregnancy.

### Relation to education and gestation age

Education and the gestation ages of the women at the time of registration for ANC at the clinics were not significantly associated with malaria infection. Low educational level was not associated with malaria infection. This may be attributed to massive radio and television campaigns on malaria prevention strategies and appropriate treatment options in Lagos. These campaigns are also regular features in child welfare clinics all over Lagos and are conducted in English, as well as local languages.

### Relation to insecticide spray

This is the first study to report the effect of the use of personal/household insecticide spray on malaria in pregnancy in Nigeria. The use of insecticide spray was very common among pregnant women in this study. Also, the frequency of use of these insecticide sprays was high, with 67.2% of the women using insecticide spray more than once a week. This probably accounted for its significant impact on malaria infection.

### Relation to insecticide treated nets (ITN)

Insecticide treated nets (ITN) coverage in this study population was far from the 60% target – of pregnant women sleeping under an ITN – that was set by African Heads of States at the Roll Back Malaria Summit held in Abuja in 2000 [33]. The higher prevalence of malaria in pregnant women that used an ITN as opposed to those that used any net suggested that strict adherence to sleeping under an ITN may be a challenge as there were complaints of excessive sweating (data not shown) caused by poor ventilation, which was further exacerbated by the frequent absence of public power supply running electric fans and air conditioners. Some other studies have also reported that net ownership does not necessarily translate to usage [34,35].

Despite reports that the use of ITNs substantially reduced the risk of malaria in pregnancy [36], the use of bed nets, whether treated or not, did not have a significant impact on malaria infection in this study. Marchant and co-workers [33] also reported a non-significant but modest impact of ITN on malaria in pregnancy. The suggested reasons for the conflicting results on the efficacy of ITN are: a) compliance with sleeping under a bed net; and b) the complex vector populations with exophagic\exophilic and early biting behaviour. The high rate of the use of insecticide spray in this study may also be responsible for the very low rate of the use of ITNs.

### Relation to the combination of preventive methods

The combined use of insecticide spray and ITN resulted in a reduction in malaria infection though this reduction was not significant. None of the women that used a combination of the three preventive methods had malaria infection. Education and age were significantly associated with the combined use of insecticide spray and ITN. This could be explained by the increased financial capacity to purchase insecticide sprays. The very strong association with age could be due to poor financial capacity of women in the 15–19 years of age group that may have resulted in the poor use of both strategies. Pregnant women above 24 years of age were likely to be employed thus resulting in a higher frequency of use (>80%) of this combination. Occupation was not significantly associated with the use of combination interventions. This could be the result of the challenge in grading the various professions into low- or high-income professions.

### Relation to chemoprophylaxis

The use of un-recommended malaria chemoprophylactic medications such as chloroquine (CQ) did not significantly reduce malaria infection among the pregnant women. This finding was not surprising because high CQ failure rates had been reported in Southwest Nigeria. The various CQ failure rates reported ranged from 37.9% to 59.1% [37-39]. Coulibaly *et al.*[40] also reported a failure rate of 46.7% among pregnant women treated with CQ in Burkina Faso. The current Nigerian National guideline for malaria prevention and control during pregnancy replaced CQ and pyrimethamine with SP as malaria chemoprophylaxis during pregnancy because of issues of compliance with the dosing regimen and resistance [41]. However, the use of un-recommended antimalarial medications as chemoprophylaxis, is likely to increase the risk of pregnant women having a malaria infection.

## Conclusions

Malaria prevalence in the studied population was low and this could be due to the scaling-up of malaria interventions, high competency in malaria microscopy (with improved microscopy sensitivity/specificity) and possibly low transmission due to the urban nature of the participants’ habitation. Young maternal age and non-usage of insecticide spray were important risk factors associated with malaria infection during pregnancy. The use of ITN and self-prescribed chemoprophylaxis did not significantly influence malaria in pregnancy.

The factors elucidated in this study should be articulated to effectively scale up malaria prevention strategies among pregnant women. Further, early attendance and utilisation of focused antenatal care services by all pregnant women will reduce the risk of malaria in pregnancy. This study also underscored the need for more expansive studies in different transmission settings to provide current data on risk factors for malaria in pregnancy in the context of scaled-up malaria control efforts.

## Abbreviations

ACT: Artemisinin-based combination therapy; ANC: Antenatal clinic; C.I.: Confidence interval; CQ: Chloroquine; IPTp: Intermittent preventive treatment of malaria in pregnancy; ITN: Insecticide treated net; OR: Odds ratio; P: Probability; RR: Relative risk; SP: Sulphadoxine-pyrimethamine; mL: Milliliter; μL: Microliter; %: Percent.

## Competing interests

The authors wish to declare that they have no competing interests.

## Authors’ contributions

CO and WA conceptualised, analysed and wrote the paper. Both authors read and approved the final manuscript.

## Supplementary Material

Additional file 1 Multilingual abstracts in the six official working languages of the United Nations. Click here for file
