# Supplementary material for: Factors associated with risk of malaria infection among pregnant women in Lagos, Nigeria
Source: Infect Dis Poverty. 2013 Aug 30;2:19. doi: 10.1186/2049-9957-2-19 (PMC3848704; doi:10.1186/2049-9957-2-19)
Supplement: Additional file 1 — Multilingual abstracts in the six official working languages of the United Nations. [file 2049-9957-2-19-S1.pdf]

Translation of the abstract into the six official working languages of the United Nations

العوامل المرتبطة بأخطار الإصابة بعدوى الملاريا بين النساء الحوامل في لاجوس، نيجيريا

شيميري أو أجومو و ويللنجتون أ أوييبو

الملخص

الخلفيات: تم حصر النساء الحوامل اللاتي يعشّن في مناطق الانتقال المستمر لعدوى الملاريا مثل لاجوس، نيجيريا. وتم تحديد المنطقة بأنّها منطقة خطر متزايد من آثار عدوى الملاريا. وتُعد معظم الإصابات في تلك المنطقة هي إصابات عرضية، بمعني أنّه يُمكنْ التغاضي عنها أوعدم معالجتِها، مما قد يؤدي إلى أضرار للأم ولجنينها. إنّ حقيقة تداخل الملاريا وتزايدها وشبكات المعالجة طويلة المدى لاصطياد الحشرات، ومُكافحة النواقل، والعلاج المُركب بالأرتيميسينين (ACT)، والعلاج الوقائي المُتقطع للملاريا أثناء الحمل (IPTp) باستخدام سلفادوكسين بيريميثامين (SP) يُعد من الضروري أيضاً لتحديد عوامل الخطر الكامن في مثل هذه الظروف. وتهدف هذه الدراسة إلى التعرف على العوامل المُرتبطة بأخطار الإصابة بعدوى الملاريا بين النساء الحوامل في لاجوس، جنوب غرب نيجيريا.

الطرق: تم الحصول على البيانات الديموغرافية والمُمارسات الوقائية لملاريا للنساء الحوامل باستخدام استبيان مُنظم. وتم استخدام الفحص المجهري لإيجاد العلاقة بين عدوى الملاريا، وبين أنواع وكثافات الطُفيليات. كما تم استخدام عمليات حساب الخطر النسبي وتحليل الانحدار اللوجستي مُتعدد المُتغيرات وذلك لمقارنة العوامل المُرتبطة بالملاريا بين النساء الحوامل.

النتائج: تم الحصول على تفاصيل الفحص المجهري الدقيق للملاريا، والبيانات الديموغرافية وبيانات الممارسات الوقائية لعدوى الملاريا بين النساء الحوامل وذلك باستخدام استبيان مُنظم. وكان مُعدل انتشار عدوى الملاريا باستخدام عينات الدم المباشرة في 1،084 من النساء الحوامل اللاتي شاركن في الدراسة هو 7.7٪. كما شوهدت المتصورة المنجلية (بي. المنجلية) في 95.2٪ من الحالات، وقد ظهرت على شكل عدوى مُختلطة مع بي.الملاريية (3.6٪)، أو على شكل عدوى أحادية (91.6٪). وقد ارتبطت المُمارسات الوقائية لعدوى الملاريا بانخفاض ملموس (P <0.05) في عدوى الملاريا عند استخدام بخاخات الحشرات (RR = 0.36, 95 C.I. 0.24-0.54)، وعند الجمع بين استخدام بخاخات رش الحشرات والناموسيات المُعالجة بمبيدات الحشرات (ITN) (RR= 6.53, 95% C.I. 0.92-46.33). كما أنّ النوم تحت الناموسيات المُعالجة بالمبيدات الحشرية وحدها (RR = 1.07, 95% C.I. 0.55-2.09) لم يترافق مع انخفاضٍ ملحوظٍ في عدوى الإصابة بالملاريا مع الطفيليات في الدم بين المشاركات في الدراسة. حيث يكون عُمر الأم الشابة (< 20 عاماً) (RR = 2.86, 95% C.I. 1.48 – 5.50)، ولكنّه لا يُعد بريميجرافيدي (RR = 1.36, 95% C.I. 0.90-2.05)، ويصاحبه زيادة في أخطار الإصابة بعدوى الملاريا خلال فترة الحمل. وبعد حساب الانحدار اللوجستي مُتعدد المُتغيرات، فإن عمر الأم الشابة (OR = 2.61, 95% C.I. 1.13 – 6.03) ومع استخدام عمليات رش المبيدات الحشرية بالبخاخات (OR = 0.38, 95% C.I. 0.24-0.63) ارتبط ارتباطاً ملحوظاً مع ارتفاع أو انخفاض عدوى الملاريا ، وذلك على التوالي.

االاستنتاج: إن انتشار الملاريا كان منخفضاً بين النساء الحوامل اللاتي تمت دراسة حالاتهن. كما كان عمر الأم الشابة في الحالات التي لم يتم فيها رش بمبيدات الحشرات من العوامل الرئيسية المُرتبطة بزيادة خطر الإصابة بعدوى الملاريا بين النساء الحوامل في لاجوس، نيجيريا.

Translated from English version into Arabic by FREELANCEABUNAR, through

尼日利亚拉格斯孕妇感染疟疾的危险因素

Chimere O. Agomo and Wellington A. Oyibo

摘要

引言：生活在尼日利亚拉格斯这样一个疟疾稳定传播地区的孕妇，其感染疟疾的风险呈上升趋势。在该地区，多数感染是无症状的，这意味着这些感染被忽视、未经治疗，对母亲和胎儿造成更大的损害。长效杀虫剂处理蚊帐、媒介控制、青蒿素联合疗法（ACT）和使用周效磺胺-乙胺嘧啶进行妊娠期间歇性预防治疗（IPTp）等疟疾防治措施的推广实施，必须掌握不同情况下的疟疾感染影响因素。本研究拟确定尼日利亚西南地区的拉格斯孕妇感染疟疾的危险因素。

方法：使用结构式问卷调查孕妇的人口信息和疟疾预防措施。利用显微镜检查疟疾感染情况、鉴别疟原虫种类和确定疟原虫密度。采用了相对风险和多因素 Logistic 回归分析比较孕妇感染疟疾的危险因素。

结果：孕妇的镜检结果、个人信息和疟疾预防措施等相关信息均通过结构式问卷获得。外周血涂片检查了 1084 位参与研究的孕妇，疟疾总感染率为 7.7%。恶性疟共占 95.2%，其中恶性疟原虫与三日疟原虫混合感染占 3.6%，单纯恶性疟原虫感染占 91.6%。与疟疾感染率显著降低（P<0.05）相关的预防措施为杀虫剂喷洒(RR = 0.36, 95 C.I. 0.24-0.54)和杀虫剂喷洒结合使用杀虫剂浸泡蚊帐（ITN）(RR= 6.53, 95% C.I. 0.92-46.33)。仅使用杀虫剂浸泡蚊帐不会显著降低疟疾带虫率(RR = 1.07, 95% C.I. 0.55-2.09)。育龄妇女<20 岁与孕期疟疾感染风险增加因素呈相关(RR = 2.86, 95% C.I. 1.48 – 5.50)，但初产妇与此不相关(RR = 1.36, 95% C.I. 0.90-2.05)。多因素 logistic 回归分析结果显示，年轻孕妇的年龄、使用杀虫剂喷洒分别是疟疾感染风险增加和降低的相关因素。

结论：在所研究的孕妇中疟疾感染率低。在尼日利亚拉格斯的孕妇中，年轻孕妇的年龄、不进行杀虫剂喷洒是疟疾感染风险增加的主要相关因素。

Translated from English version into Chinese by Yang Pin, through

CHINESE CENTER FOR DISEASE CONTROL AND PREVENTION
NATIONAL INSTITUTE FOR PARASITIC DISEASES

Facteurs associés au risque de paludisme chez les femmes enceintes au Lagos, Nigeria

Chimere O. Agomo et Wellington A. Oyibo

Résumé

Contexte : Les femmes enceintes vivant dans une zone de transmission stable du paludisme comme le Lagos, au Nigeria, ont été identifiées comme étant exposées à un risque accru des effets de l'infection du paludisme. Dans cette zone la plupart des infections sont asymptomatiques, ce qui signifie qu'elles sont négligées et traitées au détriment de la mère et son fœtus. La réalité des interventions contre l'augmentation du paludisme avec des moustiquaires imprégnées d'insecticide de longue durée, la lutte antivectorielle, la thérapie combinée artémisinine (ACT) et le traitement préventif intermittent du paludisme de la grossesse (TPI) en utilisant la sulfadoxine pyriméthamine (SP), c'est qui est également essentiel pour déterminer les facteurs de risque en jeu dans ce genre de circonstances. Cette étude visait à identifier les facteurs associés à un risque d'infection par le paludisme chez les femmes enceintes au Lagos, Nigeria du Sud-ouest

Méthodes : Les données démographiques et les pratiques de prévention du paludisme des femmes enceintes ont été capturées à l'aide d'un questionnaire structuré. Pour établir l'infection palustre a été utilisée la microscopie, l'identification des espèces et la densité parasitaire. Le risque relatif et l'analyse de régression logistique multivariée ont été utilisés pour comparer les facteurs associés au paludisme chez les femmes enceintes.

Résultats : les détails de microscopie du paludisme, les données démographiques et les pratiques de prévention du paludisme chez les femmes enceintes ont été obtenues à l'aide d'un questionnaire structuré. La prévalence du paludisme en utilisant du sang périphérique à partir de 1.084 femmes enceintes ayant participé à l'étude était de 7,7%. *Plasmodium falciparum* (*P. falciparum)* a été observé dans 95,2% des cas soit comme une infection mixte par P. malariae (3,6%) soit comme une infection mono (91,6%). Les pratiques de prévention du paludisme associées à une réduction significative (P <0,05) de l'infection du paludisme a été l'utilisation d'insecticides spray (RR = 0,36, IC 95 de 0,24 à 0,54), et l'utilisation combinée de d'insecticides et des moustiquaires imprégnées d'insecticide (MII) (RR = 6,53, IC 0,92 à 46,33 95%). Ne dormir qu'avec le ITN seulemenr (RR = 1,07, IC 0,55 à 2,09 95%) n'a pas été associée à une réduction significative de l'infection du paludisme chez les participantes à l'étude avec la parasitémie du paludisme. Le jeune âge maternel (<20 ans) (RR = 2,86, 95% C.I. 1,48 - 5,50), mais pas primipares (RR = 1,36, 95% CI 0,90 - 2,05), a été associée à un

risque accru d'infection du paludisme pendant la grossesse. Après une régression logistique multivariée, le jeune âge de la mère (OU = 2,61, 95% C.I.1, 13 - 6,03) et l'utilisation d'insecticide spray (OU = 0,38, 95% C.I. 0,24 -0,63) étaient associés à une augmentation et à une réduction de l'infection palustre, respectivement.

Conclusion: La prévalence du paludisme était faible chez les femmes enceintes étudiées. Le jeune âge de la mère et la non-utilisation d’insecticides spray ont été les principaux facteurs associés à un risque accru d'infection par le paludisme chez les femmes enceintes au Lagos, Nigeria

Translated from English version into French by Angie Garbarino, through

TRANSLATORS
WITHOUT BORDERS

Факторы риска заражения малярийной инфекции среди беременных женщин в Лагосе, Нигерия

Чимере О. Агомо и Уэлингтон А. Ойибо

Резюме

История вопроса: Беременные женщины, проживающие в такой зоне стабильной передачи малярийной инфекции, как Лагос, Нигерия, имеют повышенный риск заражения этим заболеванием. На данной территории большинство инфекций протекают бессимптомно, что зачастую приводит к отсутствию адекватного лечения в ущерб матери и плоду. Большее количество мер по борьбе с малярией, использование обработанных инсектицидами сеток против насекомых, борьба с переносчиками инфекции, основанная на артемизинине комбинированная терапия (АКТ) и периодическая профилактика заражения малярией в период беременности с помощью сульфадоксина пириметамина (СП), который также играет важную роль при определении факторов риска с учетом сложившихся обстоятельств. Данное исследование было направлено на выявление факторов, связанных с риском малярийной инфекции у беременных женщин Лагоса, юго-западная часть Нигерии.

Методология: Сбор демографических данных и информации о практиках предупреждения заражения малярией среди беременных женщин осуществлялся с помощью заполнения специально составленного опросного листа. Микроскопические исследования использовались для определения малярийной инфекции, идентификации видов и плотности паразитов. Анализ относительных рисков и множественной логической регрессии применялся для сопоставления факторов, связанных с распространением малярийной инфекции среди беременных женщин.

Результаты: Сбор данных микроскопического анализа, демографических данных и информации о практиках предупреждения заражения малярией среди беременных женщин осуществлялся с помощью заполнения специально составленного опросного листа. Заболеваемость малярией среди 1084 женщин, у которых был взят анализ периферической крови, составила 7,7%. *Plasmodium falciparum* (*P. falciparum*) был выявлен в 95,2% случаев в качестве микст-инфекции с *P. malariae* (3,6%) или моноинфекции (91,6%). Практики предупреждения заражения малярией, связанные с существенным сокращением (P<0.05) числа зараженных, предполагали использование

спреев против насекомых (RR = 0,36, 95 C.I. 0,24-0,54) и одновременное использование спреев против насекомых и обработанных инсектицидами сеток против насекомых (RR= 6,53, 95% C.I. 0,92-46,33). Только сон под такой сеткой (RR = 1,07, 95% C.I. 0,55-2,09) не показал значительного сокращения числа зараженных малярией среди участников исследования. Беременные женщины молодого возраста (<20лет)(RR = 2,86, 95% C.I. 1,48 – 5,50), за исключением случаев раннего материнства (RR = 1,36, 95% C.I. 0,90-2.05), ассоциировались с повышенным риском заражения малярийной инфекцией в период беременности. После множественной логической регрессии беременность в молодом возрасте (OR = 2,61, 95% C.I. 1,13 – 6,03) и использование спрея против насекомых (OR = 0,38, 95% C.I. 0,24-0,63) ассоциировались с ростом и сокращением числа случаев заражения малярией соответственно

Заключение: Заболеваемость малярией была достаточно низкой в изученной группе беременных женщин. Беременность в молодом возрасте и неиспользование спрея против насекомых стали основными факторами повышенного писка заражения малярией среди беременных женщин в Лагосе, Нигерия.

Translated from English version into Russian by Irina Zayonchkovskaya, through

TRANSLATORS
WITHOUT BORDERS

Factores asociados con el riesgo de infección de malaria entre mujeres embarazadas en Lagos, Nigeria

Chimere O. Agomo y Wellington A. Oyibo

Resumen

Antecedentes: Las mujeres embarazadas que viven en un área de transmisión estable de malaria como Lagos, Nigeria han sido identificadas como en mayor riesgo de los efectos de la infección por malaria. En esta área, la mayoría de las infecciones son asintomáticas lo que significa que son ignoradas y no tratadas en detrimento de la madre y su feto. La realidad es que la ampliación proporcional de las intervenciones de malaria con mosquiteros tratados con insecticidas de larga duración, vector de control, tratamiento combinado con artemisinina (ACT, por sus siglas en inglés) y el tratamiento preventivo intermitente de la malaria durante el embarazo (IPTp) con sulfadoxina-pirimetamina (SP) también resulta esencial para determinar los factores de riesgo en juego en este tipo de circunstancias. Este estudio tenía como fin identificar los factores asociados con el riesgo de infección de malaria en mujeres embarazadas en Lagos, al suroeste de Nigeria.

Métodos: se recolectó información demográfica y prácticas de prevención de la malaria de las mujeres embarazadas mediante un cuestionario estructurado. Se utilizó la microscopía para establecer la infección por malaria, identificación de la especie y densidad de parásitos. Se usaron análisis de riesgo relativo y de regresión logística multivariable para comparar los factores asociados con la malaria en las mujeres embarazadas.

Resultados: Los detalles microscópicos de la malaria, la información demográfica y las prácticas de prevención de la malaria en mujeres embarazadas se obtuvieron usando un cuestionario estructurado. La prevalencia de la malaria usando sangre periférica de 1.084 mujeres embarazadas que participaron en el estudio fue del 7,7%. Se detectó *Plasmodium falciparum* (*P. falciparum)* en el 95,2% de los casos ya sea en infección mezclada con *P. malarie* (3,6%) o como una infección única (91,6%). Las prácticas de prevención de la malaria asociadas con una reducción significativa (P<0,05) en la infección de malaria fueron el uso de insecticida en espray (RR = 0,36, 95 C.I. 0,24-0,54), y el uso combinado de insecticida en espray y mosquiteros tratados con insecticidas (ITN) (RR= 6,53, 95% C.I. 0,92-46,33). Dormir bajo un mosquitero tratado con insecticida (ITN) solamente (RR = 1,07, 95% C.I. 0,55-2,09) no se asoció con una reducción significativa de la infección de malaria

entre los participantes del estudio con malaria parasitaemia. La edad materna temprana (<20 años) (RR = 2,86, 95% C.I. 1,48 – 5,50), pero no la primera gestación (RR = 1,36, 95% C.I. 0,90-2,05), se asoció a un mayor riesgo de malaria durante el embarazo. Después de una regresión logística multivariable, la edad materna temprana y el uso de insecticida en espray (OR = 0,38, 95% C.I. 0,24-0,63) se asoció con un aumento y una reducción en la infección de malaria, respectivamente,

Conclusión: La prevalencia de malaria fue baja entre las mujeres embarazadas estudiadas. La edad materna temprana y el no uso de insecticida en espray fueron los principales factores asociados con un mayor riesgo de infección de malaria entre mujeres embarazadas en Lagos, Nigeria.

Translated from English version into Spanish by Alorcalopez, through

TRANSLATORS WITHOUT BORDERS
